# A rare case of gastroenteritis due to *salmonella enterica* serovar Infantis in South India

**DOI:** 10.3389/fpubh.2026.1874658

**Published:** 2026-09-18

**Authors:** S. C. Shruthi Shree, Yogeesh D. Maheshwarappa, Mahadevaiah Neelambike Sumana, Deepashree Rajshekar, Supreeta R. Shettar, G. K. Megha, Veerabhadraswamy G. S., Chinchana Shylaja Eshwarappa, Morubagal Raghavendra Rao, Vidhyavathi B. Chitharagi

**Affiliations:** Department of Microbiology, JSS Medical College and Hospital, JSS Academy of Higher Education and Research, Mysuru, India

**Keywords:** food-borne pathogen, gastroenteritis, non-typhoidal salmonella, salmonella enterica serovar Infantis, zoonotic infection

## Abstract

*Salmonella enterica* serovar Infantis is an emerging non-typhoidal *Salmonella* (NTS) serovar increasingly associated with foodborne gastroenteritis. Human infections caused by this serovar remain underreported in India. We report a case of acute gastroenteritis caused by *Salmonella enterica* serovar Infantis in a 53-year-old male from South India. The patient presented with acute-onset watery diarrhoea for six days associated with intermittent fever and recent outside food consumption. Stool culture yielded non-lactose fermenting colonies identified by automated identification using the VITEK-2 Compact system as *Salmonella* group, which was subsequently confirmed as *Salmonella enterica* serovar Infantis by the National Institute for Research in Bacterial Infections, Kolkata, India. Antimicrobial susceptibility testing revealed susceptibility to ceftriaxone, cefepime, carbapenems, ciprofloxacin, and trimethoprim/sulfamethoxazole, while resistance to aminoglycosides and cefuroxime-axetil was observed. The patient improved clinically with ceftriaxone therapy and supportive management. This case highlights the emerging clinical importance of *S. Infantis* in India and underscores the necessity of routine serotyping, antimicrobial susceptibility testing, and strengthened surveillance to monitor evolving resistance patterns and public health risks associated with NTS infections.

## Introduction

*Salmonella enterica* serovar Infantis (*S. Infantis*) is a non-typhoidal *Salmonella* (NTS) serovar that has emerged over the last two decades as a major cause of food-borne gastroenteritis and invasive infections worldwide (1). It is particularly linked to the escalation of poultry production. It is a motile, Gram-negative, facultatively anaerobic bacillus that produces non-lactose-fermenting colonies and H_2_S on selective enteric media (2). Historically, *S. Infantis*, considered one of many NTS serovars causing sporadic disease, has gained prominence because of the global expansion of a few successful clonal lineages that are highly adapted to broiler chickens and their production environment, resulting in frequent contamination of poultry meat and dissemination along the food chain into the human population (2–5).

In humans, *S. Infantis* is mainly transmitted through contaminated chicken meat and related products, but also through contact with infected animals, contaminated environments, and occasionally through healthcare-associated exposure (4, 6). Infection typically presents as acute, self-limiting gastroenteritis with fever, abdominal pain, watery or inflammatory diarrhea, and vomiting beginning 6–72 h post-ingestion (1, 4). Although many cases are self-limiting, *S. Infantis* has been implicated in community and institutional outbreaks with substantial morbidity, including among the individuals aged >60 years in long-term care and hospitalized patients, where about one-third of confirmed cases require hospitalization, some needing ICU care or resulting in death, especially for multidrug-resistant strains (2, 4). In vulnerable hosts such as infants, older adults, and those with comorbidities like malignancy, diabetes, chronic kidney disease, HIV, or splenectomy, it causes invasive disease with bacteremia and focal infections, including urinary tract infection, osteoarticular infection, and sepsis, as seen in reports of relapsing urinary tract infection in immunocompromised individuals (7–9).

*S. Infantis* harbors the core *Salmonella* virulence repertoire, including *Salmonella* pathogenicity islands SPI-1 and SPI-2 encoding type III secretion systems that mediate intestinal epithelial invasion, survival in macrophages, and systemic spread (1–13). A striking feature of many contemporary *S. Infantis* clones is the presence of a large pEsSI-like conjugative megaplasmid that confers virulence, fitness, stress-tolerance, biofilm-formation, and multiple antimicrobial resistance determinants, including extended-spectrum β-lactamases like blaCTX-M, tetracyclines, sulphonamides, trimethoprim, and aminoglycosides, producing a multidrug-resistant (MDR) phenotype that complicates severe infection management (14, 15).

Surveillance data from several continents identify *S. Infantis* among the leading NTS serovars from poultry, meat, and human samples, with clonal MDR spread across farms, slaughterhouses, and trade networks, increasing resistance rates and treatment failure risks in invasive disease, globally, it shows sharp rise such as a 167% increase in US human infections (2001–2016), dominance in EU broilers (46%−57%) and broiler meat (37%−51%), and outbreaks like Germany's 2013 chicken-linked event (267 cases) and the persistent US REPJFX01 MDR strain. In India, no comprehensive national surveillance data on the total number of *Salmonella enterica* serovar Infantis cases reported nationwide exists, as non-typhoidal *Salmonella* infections are underreported and not systematically tracked nationwide. In a 1993–1996 study of 1,025 hospitalized children with diarrhea in Calcutta, 14 cases (8.9% of 157 *Salmonella* isolates) were identified as *S. Infantis* (16, 17).

In India, the true prevalence of *S. Infantis* remains uncertain because non-typhoidal *Salmonella* infections are underreported and routine serotyping is not consistently performed across healthcare settings. Earlier studies from Kolkata identified *S. Infantis* among hospitalized children with diarrhea (16), while more recent reports from South India have demonstrated the emergence of poultry-associated *S. Infantis* strains possessing important virulence factors and antimicrobial resistance determinants (17, 18). These findings suggest that the organism may be under-recognized rather than truly rare because of limited epidemiological surveillance and inadequate laboratory characterization. Therefore, the present case report aims to describe the clinical presentation, microbiological identification, antimicrobial susceptibility profile, and public health significance of *Salmonella enterica* serovar Infantis causing acute gastroenteritis in South India. By documenting this microbiologically confirmed case, we seek to contribute to the limited clinical evidence available from India, emphasize the importance of routine serotyping and antimicrobial susceptibility testing, and strengthen awareness of this emerging non-typhoidal *Salmonella* serovar among clinicians, microbiologists, and public health professionals.

## Case presentation

A 53-year-old male patient presented with a history of left total knee replacement (TKR) conducted 1 month prior, a fall sustained 3 years earlier, and renal tubular acidosis detected 3 months ago, presented with complaints of loose stools for 6 days and a history of intermittent low- to moderate-grade fever, 4 days before admission. He was apparently asymptomatic until 6 days before presentation, when he developed acute-onset diarrhea, initially 6–7 episodes per day, progressively increasing to 10–12 episodes per day. The stools were watery, foul-smelling, non-bloody, and devoid of mucus.

There was no history of abdominal pain, vomiting, decreased urine output, jaundice, or weight loss. There was no history suggestive of urinary tract infection, such as increased frequency, dysuria, or urinary dribbling; however, the patient complained of a poor urinary stream. A history of recent outside food consumption and travel was present. Additionally, the patient reported reduced sleep over the past month, raising concern for possible psychological stress. There was no history of diabetes mellitus, hypertension, epilepsy, tuberculosis, bronchial asthma, or other chronic illnesses.

On examination, the patient was conscious, cooperative, and oriented to time, place, and person. He was febrile and hemodynamically stable, with a pulse rate of 78 beats/min (regular), blood pressure of 118/63 mmHg, respiratory rate of 18 cycles/min, and oxygen saturation of 98% on room air. There was no pallor, icterus, cyanosis, clubbing, or peripheral lymphadenopathy, and the tongue was moist. Cardiovascular examination revealed normal heart sounds with no added sounds. Respiratory system examination showed bilaterally equal air entry with no adventitious sounds. Abdominal examination revealed a soft abdomen with mild periumbilical tenderness, with no guarding and rigidity, organomegaly, or free fluid. Bowel sounds were present. Neurological examination demonstrated intact higher mental functions with no focal neurological deficits. On admission, a provisional diagnosis of acute gastroenteritis was made, and a stool sample was sent for microscopic examination and culture. The patient was empirically started on ceftriaxone (injection) 1 g BD (bis in die) to cover common enteric Gram-negative pathogens, and metronidazole (injection) 500 mg TID (ter in die) to cover protozoans. Details of the laboratory and practical findings were given in Table 1.

**Table 1:** Physical and systemic examinations and radiological and laboratory findings.

Parameter	Observation
Physical examination	
Blood pressure	118/63 mmHg
Pulse rate	78 beats/min
SPO_2_	91%
Febrile status	febrile
Systemic examination	
Cardiovascular system	S1 and S2
Respiratory system	B/LNVBS (bilateral normal vesicular breath sounds)
Per-abdomen examination	Soft, periumbilical, tenderness +
Central nervous system	Conscious, oriented.
Radiological findings	
Chest X-ray	No radiological abnormality
Laboratory findings	
Hemoglobin (Hb)	11.8 g/dl
Total leucocyte count (TLC)	5,070 cells/cumm
Neutrophils	65.8
Liver function test	
Bilirubin direct	0.18 mg/dl
Ast (Sgot)-aspartate aminotransferase	28 U/L
Ast (Sept)- alanine aminotransferase	25 U/L
Alkaline phosphatase (ALP)	54 U/L
Gamma-glutamyl transferase (GGT)	12 U/L
Renal function test	
Urea	16 mg/dl
Creatinine	0.92 mg/dl
Uric acid	4.2 mg/dl
Sodium	138 mEq/L
Potassium	4.5 mEq/L
Chloride	102 mEq/L
Albumin	3.9 g/dl
Total proteins	6.6 g/dl
Ultrasound sonography (USG)	
Liver	Normal in size, normal echotexture, portal vein, and CBD, no focal lesion, intrahepatic biliary radicals are not dilated
Gallbladder	Contracted
Pancreas	The head and proximal body of the pancreas appear normal; excessive bowel gas shadows obscure the rest of the pancreas
Spleen	Normal in size and Echotexture
Kidney	Normal in size, shape and position, corticomedullary differentiation is maintained, no calculi/hydronephrosis
Urinary bladder	Minimally distended
Urine routine	
Urine Color	Pale yellow
Urine Ph	7
Urine clarity	Clear
Urine specific gravity	1.006
Urine-Sugar (Glucose)	Negative
Urine-albumin (Protein)	Negative
Urine-ketone bodies	Negative
Urine urobilinogen	Negative
Urine nitrates	Negative
Urine leucocytes	Negative
Urine bilirubin	Negative

Hematological investigations revealed mild anemia with a hemoglobin level of 11.8 g/dL, reduced packed cell volume (36.7%), and red blood cell count (4.02 million/cumm). Red cell indices were within normal limits (MCV 91.3 fL, MCH 29.4 pg, MCHC 32.2 g/dL, RDW 12.8%). Peripheral blood smear examination showed normocytic normochromic erythrocytes, normal leukocyte morphology and distribution, and adequate platelet counts, consistent with normocytic normochromic anemia. The reticulocyte count was 1.12%, within normal limits, indicating the absence of a compensatory erythropoietic response.

The total leukocyte count was 5,070 cells/cumm with a normal differential and absolute counts (ANC 3,340/μL; ALC 1,260/μL). Platelet count was 2.54 lakh/cumm, within the normal range. Notably, the erythrocyte sedimentation rate (ESR) was markedly elevated at 60 mm in the first h, suggestive of an underlying inflammatory or chronic disease process. Biochemical investigations revealed normal random blood glucose (92 mg/dL), renal function parameters (urea 16 mg/dL, creatinine 0.92 mg/dL), liver function tests, serum electrolytes, uric acid, serum proteins, albumin, and albumin-globulin ratio, with no evidence of hepatic or renal dysfunction.

Urine microscopy and culture results were within normal limits. Stool microscopic results revealed moderate inflammatory cells, no RBCs (Red blood cells), and no parasitic forms, but stool culture revealed non-lactose fermenting colonies on MacConkey agar and black colonies on HEA (Hektoen enteric agar). Culture microscopy revealed motile, Gram-negative bacilli, which produced catalase but not cytochrome oxidase. The bacteria did not produce indole, urea was not hydrolysed, but citrate was utilized. They reduced nitrate to nitrites and fermented glucose with gas production. The methyl red test was positive, but the Voges-Proskauer test was negative. The Triple Sugar Iron tube produced an alkaline slant and acid butt with abundant hydrogen sulfide, thus making differentiation between *Salmonella* Paratyphi B and *Salmonella* Typhimurium challenging. Additionally, an automated identification system (Vitek-2 compact system version: 9.04.4, bioMérieux, France) identified the isolate as the *Salmonella* group.

The antimicrobial susceptibility testing (AST) was conducted using the Vitek-2 compact system version 9.04.4 (bioMérieux, France) using an N-405 card. The version of CLSI used for MIC interpretation is a Copy of Global CLSI (2020), 31^st^ edition. The isolate was sensitive to ceftriaxone, piperacillin/tazobactam, cefaperazone/sulbactam, cefepime, ciprofloxacin, gentamicin, ertapenem, imipenem, meropenem, amoxicillin/clavulanic acid, and Trimethoprim/ Sulfamethoxazole. Still, it was resistant to gentamicin, amikacin, cefuroxime and cefuroxime-axetil. The results of the Vitek-2 compact system were confirmed by the Kirby-Bauer disk-diffusion method. Quality control for both the Vitek-2 and disk diffusion methods was routinely performed in accordance with standard operating protocols guided by the M-100 CLSI (Clinical and Laboratory Standards Institute) 30th edition. Additionally, the isolate was forwarded to the National Institute for Research in Bacterial Infections (Formerly known as the National Institute of Cholera and Enteric Diseases (NICED) in Kolkata, West Bengal, India, for speciation and serotyping. The NICED/NIRBI report identified the isolate as *Salmonella enterica* serovar Infantis. The AST pattern, along with minimum inhibitory concentrations, is shown in Table 2.

**Table 2:** Antimicrobial susceptibility pattern of *Salmonella group*.

Antimicrobial agent	MIC in μg/ml	Interpretation
Cefuroxime	4	S
Cefuroxime/axetil	4	R
Ceftriaxone	≤ 0.25	S
Cefepime	≤ 0.12	S
Cefaperazone/sulbactam	≤ 8	S
Piperacillin/tazobactam	≤ 4	S
Amoxicillin/clavulanic acid	≤ 2	S
Ertapenem	≤ 0.25	S
Imipenem	≤ 0.25	S
Meropenem	≤ 0.25	S
Amikacin	≤ 1	R
Gentamicin	≤ 1	R
Ciprofloxacin	≤ 0.06	S
Trimethoprim/sulfamethoxazole	≤ 20	S
Tigecycline	≤ 0.5	S
Colistin	≤ 0.5	I

S, Sensitive; R, Resistant.

Once gastroenteritis was confirmed to be due to NTS (*Salmonella enterica* serovar Infantis), which was sensitive to ceftriaxone, ceftriaxone was continued, and metronidazole was discontinued. By the fourth day of admission, the patient was symptomatically better and afebrile, with two episodes of loose stools reported. CBC, LFT, and PT/INR showed an improving trend. As the patient complained of a poor urinary stream, Uroflowmetry was done and advised conservative management. Additionally, a psychiatrist was consulted because of insomnia, and their advice was followed. On the 5th day of admission, the patient was symptomatically better. Hence, he was discharged with the following advice.

The patient was advised to continue medications as prescribed. An oral antibiotic (cefixime 200 mg) was given in a dose of 1-0-1 for 7 days. A proton pump inhibitor (pantoprazole 40 mg) was advised once daily before food for 7 days. An antidiarrheal agent (Racecadotril 100 mg) was prescribed to be taken as needed in case of diarrhea. A combination of an alpha-blocker and 5-alpha reductase inhibitor (tamsulosin 0.4 mg with dutasteride) was advised once daily at bedtime for a duration of 1 month. A combination antidepressant and anxiolytic agent was prescribed once daily at bedtime for 7 days in view of associated insomnia/anxiety. The patient was instructed to adhere to the prescribed regimen and complete the full course of therapy. Additionally, the patient was advised to follow up in the Medicine outpatient department with the final stool culture and sensitivity reports. A review in the Psychiatry outpatient department was also recommended for further follow-up. Additionally, the patient was instructed to attend the Urology outpatient department after 1 month for repeat uroflowmetry and further evaluation.

## Discussion

*Salmonella enterica* serovar Infantis is an emerging non-typhoidal *Salmonella* (NTS) serovar that has gained increasing global attention because of its association with poultry, foodborne outbreaks, and the emergence of multidrug-resistant (MDR) lineages (4, 7, 13). The present case describes microbiologically confirmed *S. Infantis* gastroenteritis in a 53-year-old man from South India who presented with acute watery diarrhea and fever following recent consumption of outside food. The patient recovered completely following culture-guided antimicrobial therapy with ceftriaxone and supportive management, highlighting the importance of timely microbiological diagnosis in patients presenting with acute gastroenteritis.

*S. Infantis* remains an under-recognized serovar in India because routine serotyping is not performed in many diagnostic laboratories. Consequently, infections are often reported simply as non-typhoidal *Salmonella*, limiting epidemiological surveillance and obscuring the true burden of this emerging pathogen (16–18). Identification of the isolate through automated culture methods followed by serotype confirmation at the National Institute for Research in Bacterial Infections (NIRBI), Kolkata, enabled accurate diagnosis and contributed valuable epidemiological data from South India.

The diagnostic approach adopted in this case illustrates the value of conventional stool culture complemented by automated identification and reference laboratory confirmation. Stool microscopy demonstrated inflammatory cells without parasitic forms, while culture yielded non-lactose-fermenting, hydrogen sulfide-producing colonies characteristic of *Salmonella*. Because biochemical reactions alone could not reliably differentiate among certain *Salmonella* serovars, automated identification using the VITEK-2 Compact system and subsequent serotyping at the reference laboratory were essential for definitive identification. This case, therefore emphasizes that laboratories should consider referral of unusual non-typhoidal *Salmonella* isolates for serotyping whenever feasible, particularly in regions where surveillance data are limited.

In this case, the patient did not develop invasive infection; his recent hospitalization for total knee replacement, together with recent outside food consumption, represented potential predisposing factors for acquisition of infection. Fortunately, early diagnosis and prompt initiation of appropriate antimicrobial therapy may prevent progression to severe disease. Clinicians should remain aware that while most *S. Infantis* infections are self-limiting, invasive manifestations, including bacteraemia, urinary tract infection, osteoarticular infection, meningitis, and sepsis have been reported, particularly among, immunocompromised, and medically vulnerable individuals (7–9). Early recognition of patients at risk for invasive disease is therefore essential to guide appropriate investigations and treatment.

The antimicrobial susceptibility profile observed in the present isolate differs from the multidrug-resistant phenotype increasingly reported worldwide. Our isolate remained susceptible to ceftriaxone, cefepime, carbapenems, ciprofloxacin, and trimethoprim-sulfamethoxazole, allowing successful treatment with ceftriaxone. In contrast, recent international studies have documented the widespread dissemination of *S. Infantis* strains carrying pESI-like megaplasmids that encode resistance to extended-spectrum β-lactams, tetracyclines, sulphonamides, aminoglycosides, and other antimicrobial agents (3, 4, 7, 13, 14). The absence of extensive multidrug resistance in our isolate may reflect regional differences in circulating clones or the absence of these resistance plasmids. Nevertheless, the continuing global expansion of MDR *S. Infantis* highlights the need for routine antimicrobial susceptibility testing to ensure appropriate therapy and to monitor evolving resistance trends.

The antimicrobial susceptibility testing (AST) of our *Salmonella enterica* serovar Infantis isolate demonstrated susceptibility to third-generation cephalosporins (ceftriaxone), aminoglycosides, and carbapenems, with resistance to ampicillin and cotrimoxazole. These findings align with earlier Indian observations. Taneja et al. (Chandigarh, India, 2014), through stool culture, serotyping, and Kirby-Bauer susceptibility testing, reported that non-typhoidal *Salmonella* isolates, including *S. Infantis*, frequently retained susceptibility to third-generation cephalosporins while showing resistance to conventional first-line drugs such as ampicillin and cotrimoxazole. The authors interpreted this as a consequence of long-standing antibiotic pressure leading to selective resistance against older agents (3, 7, 19, 20).

The clinical manifestations observed in our patient are consistent with the established virulence mechanisms of *S. Infantis*. Following ingestion of contaminated food, the organism invades intestinal epithelial cells through SPI-1-encoded type III secretion systems, inducing mucosal inflammation that results in diarrhea, fever, and abdominal discomfort. Subsequent intracellular survival mediated by SPI-2 facilitates persistence within macrophages and, in susceptible individuals, may promote systemic dissemination (11, 12). The absence of bacteraemia or extraintestinal complications in our patient likely reflects effective host immune responses together with early institution of appropriate antimicrobial therapy (25, 26).

The previously published reports demonstrate both similarities and important differences. Earlier reports from India have described uncommon invasive manifestations such as meningitis and cellulitis, whereas the present patient developed uncomplicated gastroenteritis without systemic dissemination (21–24). Likewise, recent studies from Europe and other countries have highlighted the increasing prevalence of MDR *S. Infantis* associated with poultry production and food chains (4, 7, 13), whereas our isolate retained susceptibility to several first-line agents used for severe salmonellosis. These findings suggest that although the clinical presentation was typical, the antimicrobial susceptibility profile differs from many contemporary MDR clones reported internationally, emphasizing the importance of regional surveillance (27–29).

This case has several important implications for clinical practice. Physicians should consider non-typhoidal *Salmonella*, including *S. Infantis*, in the differential diagnosis of patients presenting with acute febrile gastroenteritis following consumption of potentially contaminated food. Whenever stool culture yields *Salmonella*, serotyping and antimicrobial susceptibility testing should be performed whenever possible to facilitate accurate diagnosis, guide antimicrobial therapy, and strengthen local surveillance programmes. Increased awareness among clinicians and microbiologists may improve recognition of emerging serovars that would otherwise remain unreported.

The present report has certain limitations. As a single-case report, the findings cannot be generalized to all patients with *S. Infantis* infection. Whole-genome sequencing and molecular characterization of virulence and antimicrobial resistance determinants, including detection of pESI-like megaplasmids, were not performed. Furthermore, the precise food source could not be microbiologically confirmed, preventing definitive identification of the route of transmission.

This case highlights the emergence of *Salmonella enterica* serovar Infantis as a clinically relevant cause of gastroenteritis in India. Accurate microbiological identification, routine serotyping, and antimicrobial susceptibility testing are essential for optimal patient management and for strengthening epidemiological surveillance. Reporting such microbiologically confirmed cases will improve understanding of the evolving epidemiology of this emerging serovar and support timely public health interventions aimed at limiting the spread of antimicrobial-resistant *Salmonella*.

## Conclusion

*Salmonella enterica* serovar Infantis is an emerging NTS serovar increasingly associated with foodborne gastroenteritis. The present case emphasizes the clinical significance of this relatively underreported serovar in the Indian setting. The patient presented with acute watery diarrhea and fever following recent outside food consumption and responded favorably to ceftriaxone-based therapy after culture-guided management. Although the isolate retained susceptibility to third-generation cephalosporins, carbapenems, and fluoroquinolones, the growing global emergence of multidrug-resistant *S. Infantis* strains carrying plasmid-mediated resistance determinants remains a major public health concern. This case underscores the importance of routine stool culture, serotyping, and antimicrobial susceptibility testing for accurate diagnosis and appropriate antimicrobial therapy. Furthermore, strengthening surveillance systems and adopting a One Health approach, integrating human, animal, and environmental health, are essential to monitor the spread of resistant strains. Reporting such cases contributes valuable epidemiological data from India and aids in improving regional understanding of evolving non-typhoidal *Salmonella* infections.

## Data Availability

The original contributions presented in the study are included in the article/Supplementary material, further inquiries can be directed to the corresponding author.
